# On the Use of Phytolacca Decandra in Camp Itch

**Published:** 1864-03

**Authors:** Jno. H. Claiborne

**Affiliations:** in charge of Petersburg Hospitals.


ART. V.—*On the use of Phytolacca Decandra in Camp Itch.* By Surgeon JNO. H. CLAIBORNE, in charge of Petersburg Hospitals.

In this time of scarcity of medical supplies, I beg to call attention to two articles of indigenous growth, which I have used with success in the treatment of a disease very prevalent now in the army, viz: "Camp Itch."

These remedies are the phytolacca decandra (poke) and the broom sedge or broom straw. The botanical name for the latter, I do not know, but it is a growth that covers every impoverished and worn-out field in Eastern and Piedmont Virginia, and well known to every person. The poke is scarcely less common and equally well known. Before describing the manner of administration of either of the remedies, or detailing the circumstances under which the one or the other should be exhibited, I beg to remark that I apprehend no little confusion exists in the minds of some medical officers in regard to the precise character and classification of camp itch. It is one thing in its inception and early stage, and another as it progresses, with or without treatment, to the aggravated condition in which it is often met with. The disease which generally has been brought under my observation, and registered by medical officers as "itch," or "camp itch," is rarely *scabies*, almost never. In the treatment, therefore, which I propose, I do not wish to be understood as referring to that disease.

Camp itch, as I have observed it in its beginning, is not a disease in which the animalcule of scabies, the acarus, is ever seen. It is not even a vesicular disease. It is papular, and whilst almost a *sui generis*, is more nearly akin in my estimation, to lichen, some cases to prurigo, than to any other skin disease. When chronic, subjected to the irritation of scratching and to neglect and an hundred influences, unpropitious and aggravating, such as a soldier in camp is exposed to, it changes its character—seems sometimes pustular, sometimes vesicular, occasionally squamous—very often when about the face and neck and head, would pass for eczema impetiginodes, which it very much resembles. Now it is in the earlier and papular condition of the disease that I have found the poke root of most service. Using the root in the form of a strong decoction, as a bath, once or twice a day, then washing off with soap and water and changing the clothes frequently, I believe the disease may often be cured in a week or ten days. When scratching, or other sources of irritation, have changed the character of the disease, and particularly, if much or any inflammation of the skin attend the eruption, this treatment will give great pain—few patients can stand it, and it is in these cases I use the decoction of broom-straw root, three or four times a day, and if this gives pain, even substituting for it the decoction of slippery elm. When the irritation subsides, the decoction of poke may be used again, regulating the strength of the decoction by the smarting effect produced on the patient. With all of this, I have found it very necessary to regulate the diet of the patient, a very important indication I think—restraining him from meats of all sorts and substituting vegetable and farinaceous articles. If there be utter absence of officinal articles of the pharmacopœia, viz: arsenic, mercury, &c., I should use, and have used, with good effect, the saturated tincture of the berries of the phytolacca dec. A teaspoonful of the sat. tinct. three times a day is a good laxative, and enjoys some reputation as an alterative.

I believe the treatment which I have thus briefly indicated, will, in eight out of ten cases, effect relief, as soon as can be secured by sulphur, arsenic, or the alkaline baths, which are the routine treatment, and which are now so difficult to procure.

Of course, I should prefer, as joined to the treatment I have proposed, the constitutional remedies of mercury and arsenic, one to correct the vitiated secretions of the upper bowels and the other as alterative.

